# Agelasine D Suppresses RANKL-Induced Osteoclastogenesis via Down-Regulation of c-Fos, NFATc1 and NF-κB

**DOI:** 10.3390/md12115643

**Published:** 2014-11-24

**Authors:** Moo Rim Kang, Sun Ah Jo, Yeo Dae Yoon, Ki Hwan Park, Soo Jin Oh, Jieun Yun, Chang Woo Lee, Ki-Hoan Nam, Youngsoo Kim, Sang-Bae Han, Jiyeon Yu, Jaerang Rho, Jong Soon Kang

**Affiliations:** 1 Bio-Evaluation Center, Korea Research Institute of Bioscience and Biotechnology, 30 Yeongudanjiro, Cheongju 363-883, Korea; E-Mails: kangmr@kribb.re.kr (M.R.K.); jsunada123@naver.com (S.A.J.); yunyd76@hanmail.net (Y.D.Y); brightnessd@nate.com (K.H.P.); diatree@kribb.re.kr (S.J.O.); jyun@kribb.re.kr (J.Y.); changwoo@kribb.re.kr (C.W.L.); 2 Laboratory Animal Resource Center, Korea Research Institute of Bioscience and Biotechnology, 30 Yeongudanjiro, Cheongju 363-883, Korea; E-Mail: namk@kribb.re.kr; 3 College of Pharmacy, Chungbuk National University, 52 Naesudongro, Cheongju 361-763, Korea; E-Mails: youngsoo@chungbuk.ac.kr (Y.K.); shan@chungbuk.ac.kr (S.-B.H.); 4 Department of Microbiology & Molecular Biology, Chungnam National University, 99 Daehakro, Daejeon 305-764, Korea; E-Mails: julmooni@hanmail.net (J.Y.); jrrho@cnu.ac.kr (J.R.)

**Keywords:** agelasine D, osteoclastogenesis, c-Fos, NF-ATc1, NF-κB

## Abstract

In the present study, we investigated the effect of agelasine D (AD) on osteoclastogenesis. Treatment of bone marrow macrophages (BMMs) with receptor activator of nuclear factor κB ligand (RANKL) resulted in a differentiation of BMMs into osteoclasts as evidenced by generation of tartrate-resistant acid phosphatase (TRAP)-positive, multinucleated cells and formation of pits in calcium phosphate-coated plates. However, RANKL-induced osteoclastogenesis was significantly suppressed by AD treatment. We also confirmed the increased mRNA and protein expression of osteoclastic markers, such as TRAP, cathepsin K and matrix metalloproteinase-9, during RANKL-induced osteoclast differentiation and this was down-regulated by AD treatment. Moreover, AD treatment significantly suppressed RANKL-induced mRNA expression of DC-STAMP and OC-STAMP and cell fusion of TRAP-positive mononuclear osteoclast precursors. In addition, AD suppressed RANKL-induced expression of transcription factors, c-Fos and nuclear factor of activated T cells c1 (NFATc1), which are important transcription factors involved in differentiation of BMMs into osteoclasts. Furthermore, RANKL-induced phosphorylation of extracellular signal-related kinase (ERK) and activation of NF-κB were also inhibited by AD treatment. Collectively, these results suggest that AD inhibits RANKL-induced osteoclastogenesis by down-regulation of multiple signaling pathways involving c-Fos, NFATc1, NF-κB and ERK. Our results also suggest that AD might be a potential therapeutic agent for prevention and treatment of osteoporosis.

## 1. Introduction

Bone, a mineralized connective tissue, is a rigid yet dynamic organ. The dynamic regulation of bone mass homeostasis, which is termed bone remodeling, is mediated by two coupled processes: bone resorption by osteoclasts and bone formation by osteoblasts. Deregulation of bone remodeling can lead to various skeletal diseases, such as osteoporosis and osteopetrosis [1,2]. In addition, skeletal diseases accompanying bone loss, including osteoporosis and Paget’s disease, are characterized by progressive and/or excessive bone resorption by osteoclasts [3].

Osteoclasts are multinucleated cells differentiated from hematopoietic precursor cells of monocyte/macrophage lineage [4]. Receptor activator of NF-κB ligand (RANKL) and macrophage colony-stimulating factor (M-CSF) are two essential cytokines for osteoclastogenesis [5,6]. M-CSF is constitutively expressed by osteoblasts, whereas RANKL, a member of the tumor necrosis factor (TNF) family cytokines, is expressed by osteoblasts in response to osteotropic factors [7]. RANKL interacts with its cognate receptor, RANK, which is expressed in osteoclast precursors. The RANKL/RANK interaction recruits tumor necrosis factor receptor (TNFR)-associated factors 6 (TRAF6) close to the membrane and this evokes the activation of downstream signaling cascades, such as nuclear factor-κB (NF-κB) and mitogen-activated protein kinases (MAPKs) pathway [8]. The activation of TRAF6-dependent signaling pathways ultimately lead to induction and activation of nuclear factor of activated T cells c1 (NFATc1), a master transcription factor for osteoclast differentiation [9,10,11].

Marine sponges of the genus *Agelas* was shown to be an excellent source of novel natural compounds with various chemical structures, such as diterpene alkaloids, bromopyrrole alkaloids and glycosphingolipids [12,13,14]. Agelasine D (AD) is a diterpene alkaloid isolated from marine sponges of *Agelas* sp. [15]. Agelasine D was shown to have a variety of biological activities, including antimicrobial, antineoplastic and antifouling effects [15,16]. While searching for novel biological activities of agelasine D on immune system, we found that agelasine D exerts an inhibitory effect on osteoclast differentiation. In this study, we investigated the effects of agelasine D on osteoclastogenesis and the molecular mechanisms responsible for this effect.

## 2. Results and Discussion

### 2.1. AD Inhibits RANKL-Induced Osteoclast Differentiation

**Figure 1:**
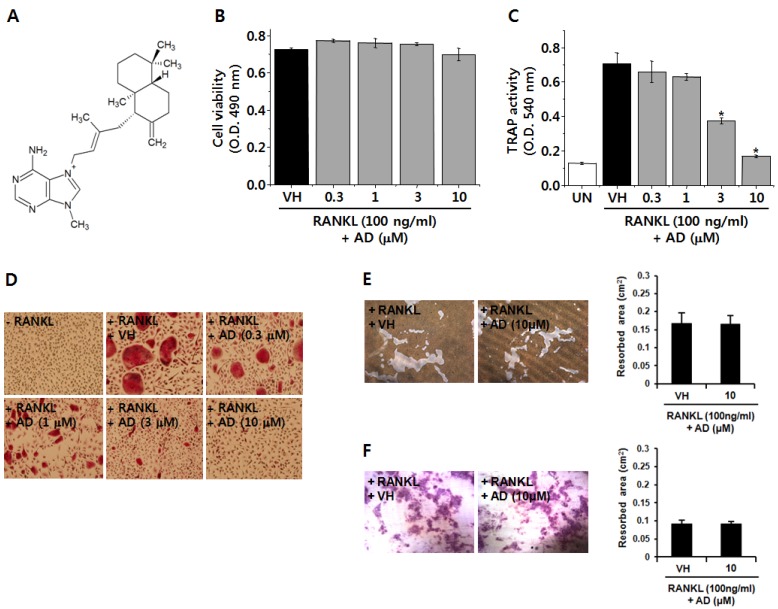
(**A**) Chemical structure of agelasine D (AD); bone marrow macrophages (BMMs) were treated with vehicle or indicated concentrations of AD in the presence of receptor activator of nuclear factor κB ligand (RANKL) and macrophage colony-stimulating factor (M-CSF) for 4 days; (**B**) Cell viability was measured by XTT assay; (**C**) Culture supernatants were mixed with chromogenic substrate containing tartrate-resistant acid phosphatase (TRAP) and the activity was determined by measuring optical density at 540 nm; (**D**) Cells were fixed with 10% formalin and stained with chromogenic substrate containing TRAP; Purified mature osteoclasts were plated on calcium phosphate-coated plates (**E**) or dentin slices (**F**) and treated with vehicle or AD (10 µM) in the presence of RANKL and M-CSF for 2 days. Pit formation was photographed under a light microscope. Each column shows the mean ± SD of triplicate determinations. Statistical significance was analyzed by one-way ANOVA and Dunnett’s *t*-test (* *p* < 0.05).

AD is a diterpene alkaloid as shown in Figure 1A. To investigate the effect of AD on osteoclastogenesis, we examined the effect of AD on RANKL-induced osteoclast differentiation in mouse bone marrow cells. AD had no cytotoxic effects on bone marrow macrophages (BMMs) differentiating into osteoclasts at concentrations used in this study (Figure 1B). To determine the effect of AD on osteoclastogenesis, we used tartrate-resistant acid phosphatase (TRAP) as a primary marker of osteoclast differentiation because TRAP is known to be highly expressed in osteoclasts [17]. As shown in Figure 1C,D, bone marrow macrophages (BMMs) differentiated into TRAP-positive multinucleated osteoclasts in the presence of RANKL and M-CSF. However, pretreatment of AD dose-dependently suppressed RANKL-induced differentiation of BMMs into osteoclast (Figure 1C,D). However, AD had no effect on bone resorption activity of mature osteoclasts (Figure 1E,F). Collectively, these results suggest that AD exerts an inhibitory effect on RANKL-induced osteoclast differentiation in BMMs.

The effect of RANKL and M-CSF is mediated by binding of these proteins to their cognate receptors, RANK and c-fms, respectively. Therefore, down-regulation of receptor expression might contribute to the reduced differentiation of BMMs into osteoclasts. To examine whether AD affects the expression of receptors for RANKL and M-CSF, we investigated the effect of AD on cell surface expression of RANK and c-fms in osteoclast precursors. As shown in Supplementary Figure 1A and Supplementary Figure 1B, AD had no effect on the cell surface expression of RANK and c-fms in RANKL-treated osteoclast precursors. These results suggest that AD might exert its inhibitory effect on osteoclast differentiation not by inhibiting the expression of receptors involved in osteoclastogenic signaling but by suppressing signaling pathways initiated from these receptors after receptor-ligand binding.

### 2.2. AD Suppresses RANKL-Induced mRNA and Protein Expression of Osteoclastic Markers

**Figure 2:**
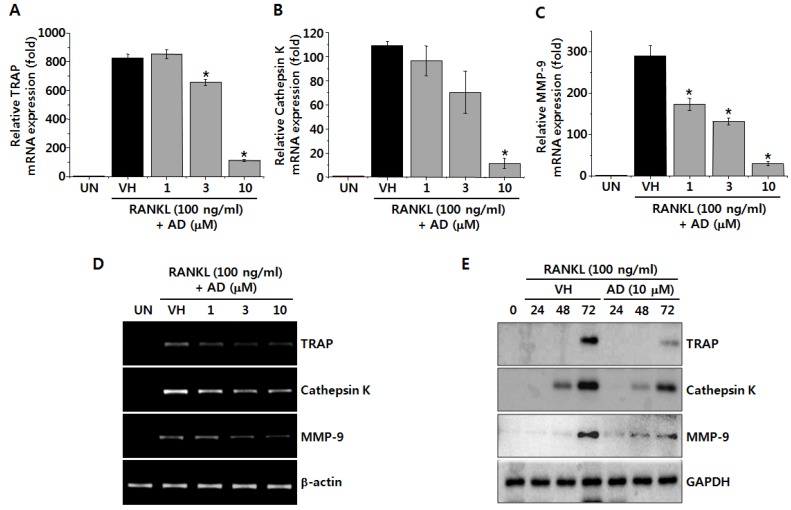
BMMs were treated with vehicle or indicated concentrations of AD in the presence of RANKL and M-CSF for 3 days. Total RNAs were isolated and the mRNA expression of TRAP (**A**), cathepsin K (**B**) and MMP-9 (**C**) was analyzed by quantitative RT-PCR. Each column shows the mean ± SD of triplicate determinations. Statistical significance was analyzed by one-way ANOVA and Dunnett’s *t*-test (* *p* < 0.05); (**D**) Gel photos of RT-PCR results; (**E**) The protein expression level of TRAP, cathepsin K and MMP-9 was analyzed by Western immunoblot analysis.

To further confirm, we examined the mRNA expression of osteoclastic markers, such as TRAP, cathepsin K and matrix metalloproteinase-9 (MMP-9), in RANKL-stimulated BMMs. In consistent with the results of TRAP assay and pit formation assay, the RANKL-induced mRNA expression of TRAP was inhibited by AD treatment in concentration-dependent manner (Figure 2A,D). Cathepsin K is a cysteine protease secreted by osteoclasts and degrades collagen and other matrix proteins during bone resorption [18]. It has also been reported that cathepsin K is involved in proteolytic processing and polarized secretion of TRAP [19]. In addition, Lotinun and coworkers reported that osteoclast-specific cathepsin K deletion stimulates bone formation [20], suggesting a critical role of cathepsin K in osteoclast function. Therefore, we examined the mRNA expression of cathepsin K and showed that AD significantly suppressed RANKL-induced mRNA expression of cathepsin K in BMMs (Figure 2B,D). MMP-9, which is highly expressed in osteoclasts, is another protease involved in the degradation of matrix proteins during bone resorption [21]. In this study, we demonstrated that the mRNA expression of MMP-9 is concentration-dependently suppressed by AD treatment in BMMs (Figure 2C,D). In addition, we also confirmed that AD treatment also suppressed the protein expression of TRAP, cathepsin K and MMP-9 in RANKL-stimulated BMMs (Figure 2E). Collectively, these results demonstrate that osteoclastic markers are down-regulated by AD treatment, confirming the inhibitory effect of AD on RANKL-induced osteoclast differentiation in BMMs.

### 2.3. AD Inhibits RANKL-Induced mRNA Expression of Fusion-Related Molecules

**Figure 3:**
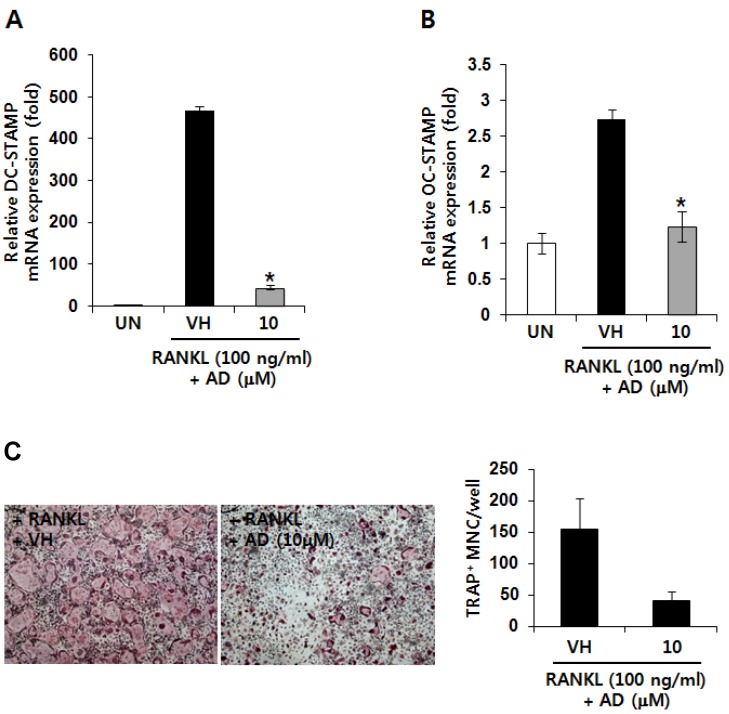
TRAP-positive mononuclear ostoclast precursors were purified and treated with vehicle or AD (10 µM) in the presence of RANKL and M-CSF for 24 h. Total RNAs were isolated and the mRNA expression of dendritic cell-specific transmembrane protein (DC-STAMP) (**A**) and osteoclast-stimulatory transmembrane protein (OC-STAMP) (**B**) was analyzed by quantitative RT-PCR; (**C**) Cell fusion of TRAP-positive mononuclear ostoclast precursors was analyzed by TRAP staining. The number of multi-nucleated osteoclasts was counted in each well. Each column shows the mean ± SD of triplicate determinations. Statistical significance was analyzed by one-way ANOVA and Dunnett’s *t*-test (* *p* < 0.05).

Cell-cell fusion is essential for generation of intact multinucleated osteoclasts and fusion-related molecules, such as dendritic cell-specific transmembrane protein (DC-STAMP) and osteoclast-stimulatory transmembrane protein (OC-STAMP), have been known to be involved in this process [22,23]. In addition, the expression of DC-STAMP and OC-STAMP is induced by RANKL-RANK signaling [22,23]. Therefore, we examined the effect of AD on the RANKL-induced mRNA expression of DC-STAMP and OC-STAMP. As shown in Figure 3A, AD treatment significantly suppressed RANKL-induced mRNA expression of DC-STAMP. Figure 3B also shows that RANKL-induced expression of OC-STAMP mRNA was down-regulated by AD treatment. To further investigate the effect of AD on cell fusion of osteoclast precursors, we performed cell fusion assay. The results presented in Figure 3C shows that AD treatment significantly attenuated RANKL-induced fusion of purified osteoclast precursors. These results suggest that the inhibitory effect of AD on RANKL-induced expression of fusion-related molecules, such as DC-STAMP and OC-STAMP, might contribute to its inhibition of RANKL-induced osteoclast differentiation. 

### 2.4. AD Negatively Regulates RANKL-Induced Expression of c-Fos and NFATc1 and Attenuates RANKL-Induced Phosphorylation of ERK

**Figure 4:**
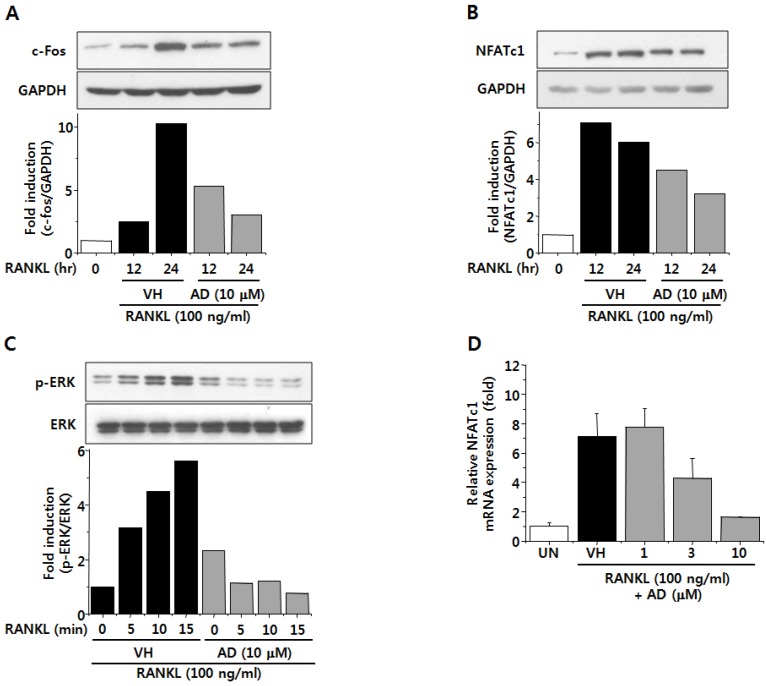
BMMs were pretreated with vehicle or indicated concentrations of AD in the presence of M-CSF for 1 h and stimulated with RANKL for indicated times. Total cell lysates were prepared and the expression of c-Fos (**A**) and nuclear factor of activated T cells c1 (NFATc1) (**B**) and the phosphorylation of extracellular signal-related kinase (ERK) (**C**) were analyzed by Western Immunoblot analysis; (**D**) BMMs were treated with vehicle or indicated concentrations of AD in the presence of RANKL and M-CSF for 3 days. Total RNAs were isolated and the mRNA expression of NFATc1 was analyzed by quantitative RT-PCR.

c-Fos and NFATc1 are important transcription factors involved in osteoclastogenesis. c-Fos is known as a key regulator of osteoclast-macrophage lineage determination and bone remodeling and mice lacking c-Fos were shown to develop osteopetrosis [24]. NFATc1, the expression of which was autoamplified by TRAF6- and c-Fos-dependent manner, is well-known as a master transcription factor for osteoclast differentiation [11]. To investigate the molecular mechanism responsible for the inhibitory effect of AD on RANKL-induced osteoclast differentiation, we analyzed the effect of AD on RANKL-induced expression of c-Fos and NFATc1. As shown in Figure 4A, c-Fos expression was increased during osteoclastogenesis induced by RANKL and M-CSF and this was suppressed by AD treatment. The protein and mRNA expression of NFATc1 was also down-regulated by treatment with AD (Figure 4B,D). These results suggest that the inhibitory effect of AD on osteoclast differentiation is mediated, at least in part, by down-regulation of c-Fos and NFATc1 in BMMs. To characterize earlier signaling events involved in the inhibitory effect of AD on osteoclastogenesis, we examined the effect of AD on RANKL-induced phosphorylation of ERK, which has been known to regulate the activity of transcription factor c-Fos [25]. Figure 4C shows that treatment of BMMs with AD suppressed RANKL-induced ERK phosphorylarion, suggesting that the inhibition of ERK phosphorylation might also contribute to the inhibitory effect of AD on osteoclastogenesis. In summary, our results suggest that AD inhibits RANKL-induced osteoclastogenesis by down-regulation of c-Fos and NFATc1, which might be preceded by attenuation of ERK phosphorylation.

### 2.5. AD Inhibits RANKL-Induced NF-κB Activation

**Figure 5:**
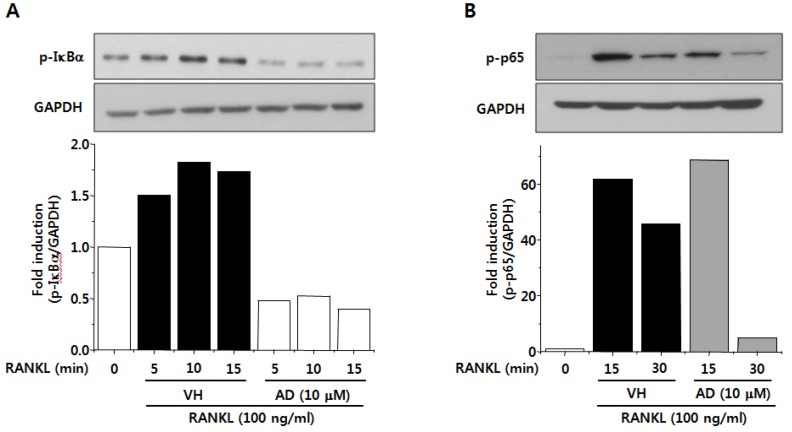
BMMs were pretreated with vehicle or indicated concentrations of AD in the presence of M-CSF for 1 h and stimulated with RANKL for indicated times. Total cell lysates were prepared and the phosphorylation of IκBα (**A**) and p65 subunit of NF-κB (**B**) were analyzed by Western immunoblot analysis. Results are representatives of more than two independent experiments.

NF-κB is a pleiotropic transcription factor involved in various biological processes. NF-κB exists in cytoplasm complexed with an inhibitory protein, called IκB in unstimulated state. IκB is phosphorylated and degraded upon activation and p65/p50 heterodimer is translocated into the nucleus and activates transcription [26]. Specifically, NF-κB is known as a key regulator of osteoclast differentiation and it has been reported that mice deficient in p50 and p52 subunits of NF-κB failed to generate mature osteoclasts and B cells [27]. In addition, it has been reported that phosphorylation of p65 subunit is required for full induction of NF-κB transcriptional activity [28]. Therefore, we examined the effect of AD on NF-κB activation by analyzing phosphorylation of IκBα and p65, which are critical steps of NF-κB activation. Figure 4A shows that the phosphorylation of IκBα was increased after RANKL treatment. However, treatment of AD dramatically inhibited RANKL-induced phosphorylation of IκBα (Figure 5A). Treatment of BMMs with RANKL also induced phosphorylation of p65 subunit and this was suppressed by AD treatment (Figure 5B). From these results, it is suggested that the inhibition of NF-κB activity might also be involved in the inhibitory effect of AD on RANKL-induced osteoclast differentiation.

## 3. Experimental Section

### 3.1. Materials

Agelasine D, which was isolated from *Agelas nakamurai*, was purchased from Alexis Biochemical Corporation (Cat. No.: ALX-350-315, Lausen, Switzerland). M-CSF and RANKL was purchased from Peprotech (London, UK). Cell Proliferation Assay Kit and TRAP staining kit was purchased from Roche Applied Science (Mannheim, Germany) and Kamiya Biomedical Company (Seattle, WA, USA), respectively. Synthetic calcium phosphate-coated plates were purchased from Cosmo Bio Co., Ltd. Antibodies for c-Fos, NFATc1, ERK, p-ERK, p-IκBα, p-p65 and GAPDH were purchased from Cell Signaling Technology (Danvers, MA, USA).

### 3.2. Bone Marrow Macrophage (BMM) Isolation and Induction of Osteoclast Differentiation

Mouse bone marrow cells were isolated from femurs and tibiae of 6~8 weeks old female C57BL/6 mice (Koatech, Pyungtaek, Gyeonggi, Korea). After lysing red blood cells, cells were incubated in α-minimal essential medium (Gibco BRL, Rockville, MD, USA) supplemented with 10% fetal bovine serum, 100 U/mL penicillin and 100 µg/mL streptomycin in the presence of M-CSF (50 ng/mL) for 3 days. Bone marrow macrophages (BMMs) were obtained by removing floating cells. For osteoclast differentiation, BMMs (4 × 10^4^ cells/well) were cultured in the presence of M-CSF (50 ng/mL) and RANKL (100 ng/mL) in 96-well plates with or without AD. After 4 days, cells were fixed with 10% formalin for 5 min and staining of TRAP-positive cells and quantitation of TRAP activity in culture supernatants were performed using TRAP staining kit (Kamiya Biomedical Company, Seattle, WA, USA) according to manufacturer’s instructions.

### 3.3. Cell Viability Assay

For cell viability assay, BMMs (4 × 10^4^ cells/well) were cultured in the presence of M-CSF (50 ng/mL) in 96-well plates with or without AD. After 4 days, XTT assay was measured using Cell Proliferation Assay Kit II (Roche). In brief, The XTT labeling mixture was prepared by mixing 50 volumes of 1 mg/mL sodium 3′-[1-(phenylaminocarbonyl)-3,4-tetrazolium]-bis(4-methoxy-6-nitro) benzene sulfonic acid hydrate (in RPMI 1640) with 1 volume of 0.383 mg/mL of *N*-methyldibenzopyrazine methyl sulfate (in PBS). This XTT labeling mixture was added to the cultures and incubated for 2 h at 37 °C. Absorbance was measured at 490 nm with a reference wavelength at 650 nm.

### 3.4. Resorption Assay

BMMs were differentiated into mature osteoclasts on collagen plates and mature osteoclasts were isolated using 0.1% type II collagen (Sigmal-Aldrich, St. Louis, MO, USA) as described previously [29]. Purified mature osteoclasts (1 × 10^4^ cells/well) were cultured in the presence of M-CSF (50 ng/mL) and RANKL (100 ng/mL) for 2 days on the Corning Osteo Assay Surface plate (Corning, Lowell, MA, USA) or a dentin slice. To detect pit formation, osteoclasts were removed using 10% sodium hypochlorite, the resorption pit was stained with 1% toluidine blue in case of dentin slice and photographed under a light microscope.

### 3.5. Cell Fusion Assay

Mouse bone marrow cells (2 × 10^6^ cells/dish) were co-cultured on UAMS32 cells (2 × 10^5^ cells/dish) with 10^−8^ M vitamin D_3_ (Sigma-Aldrich) and 10^−6^ M prostaglandin E_2_ (Sigma-Aldrich, St. Louis, MO, USA) for 6 days. TRAP-positive mononuclear osteoclast precursors were purified as described previously [30]. Purified osteoclast precursors (1 × 10^5^ cells/well) were seeded in 96-well plates and treated with vehicle of AD (10 µM) in the presence of M-CSF (50 ng/mL) and RANKL (100 ng/mL) for 24 h to induce cell fusion of osteoclast precursors. Cells were stained with TRAP and multi-nucleated osteoclasts with more than three nuclei formed by cell fusion were counted.

### 3.6. Real-Time Reverse Transcription-Polymerase Chain Reaction (RT-PCR) Analysis

Total RNAs were extracted using RNeasy Mini Kit (QIAGEN, Valencia, CA, USA) and cDNAs were synthesized using AccuPower^®^ RT PreMix (BIONEER Corporation, Daejeon, Korea). SYBR Green PCR Master Mix (Life Technologies corporation, Carlsbad, CA, USA) and ABI 7500 Sequence Detection System (Applied Biosciences, Foster City, CA, USA) were used for real-time PCR analysis. Sequences of primers used in this study are listed in Table 1. Samples were amplified by 40 cycles of 15 s denaturation at 95 °C and 1 min amplification at 60 °C. The amount of each cDNA was determined and normalized by the amount of β-actin cDNA.

**Table 1:** Primer sequences for DC-STAMP, OC-STAMP, TRAP, Cathepsin K, MMP-9 and β-actin.

Gene Name	Primer Sequence
DC-STAMP	forward: 5′-tggaggttcacttgaaactacgtg-3′ reverse: 5′-ctcggtttcccgtcagcctctctc-3′
OC-STAMP	forward: 5′-cagccacggaacacctct-3′ reverse: 5′-ggacaggctgggagaagg-3′
TRAP	forward: 5′-ctgctgggcctacaaatat-3′ reverse: 5′-ggtagtaagggctgggaag-3′
Cathepsin K	forward: 5′-aggcggctatatgaccactg-3′ reverse: 5′-ccgagccaagagagcatatc-3′
MMP-9	forward: 5′-cgtcgtgatccccacttact-3′ reverse: 5′-agagtactgcttgcccagga-3′
β-actin	forward: 5′-tggaatcctgtgcgatccatgaaa-3′ reverse: 5′-taaaacgcagctcagtaacagtccg-3′

### 3.7. Western Immunoblot Analysis

Cells were lysed with RIPA buffer (50 mM Tris-Cl [pH 8.0], 5 mM EDTA, 150 mM NaCl, 1% NP-40, 0.1% SDS and 1 mM phenylmethylsulfonyl fluoride) and cell lysates were centrifuged at 12,000 rpm for 20 min. Supernatants were collected and protein concentrations were determined using a Bio-Rad protein assay kit (Bio-Rad Laboratories, Inc., Hercules, CA, USA) according to manufacturer’s instructions. Equal amounts of proteins were resolved by 10% SDS-polyacrylamide gel electrophoresis and transferred to nitrocellulose membranes. The membranes were incubated with blocking buffer (Tris-buffered saline containing 0.2% Tween-20 and 3% non-fat dried milk) and probed with the indicated primary antibodies. After washing, membranes were probed with hoserasidh peroxidase-conjugated secondary antibodies. Detection was performed using an enhanced chemilunimescence protein (ECL) detection system (Amersham Biosciences, Little Chalfont, UK).

### 3.8. Statistical Analysis

Results are expressed as mean ± SD. One-way ANOVA and Dunnett’s *t*-test were used for multiple comparisons using GraphPad Prism (GraphPad Software, Inc., San Diego, CA, USA). The criterion for statistical significance was set at *p* < 0.05.

## 4. Conclusions

**Figure 6:**
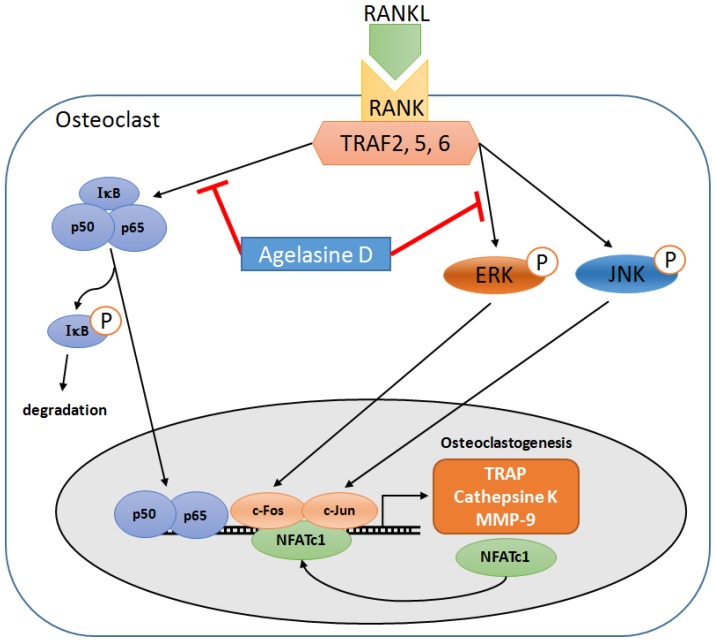
Schematic diagram of signaling pathways important for RANKL-induced osteoclastogenesis. The inhibitory effect of AD is mediated by blocking the activation of NF-κB and ERK signaling pathways and concomittant down-regulation of c-Fos and NFATc1.

In this report, we demonstrated that AD inhibits RANKL-induced differentiation of BMMs into osteoclasts and confirmed this by analyzing the expression of osteoclastic markers. We also showed that the expression and/or activation of multiple signaling molecules, including c-Fos, NFATc1, ERK and NF-κB, during RANKL-induced osteoclastogenesis, are also down-regulated by AD treatment (Figure 6). However, further studies are required to elucidate the ultimate molecular targets responsible for the inhibitory effect of AD on osteoclastogenesis. In summary, our results suggest that AD might be a therapeutic candidate for various skeletal diseases accompanying bone loss.

## Supplementary Files

Supplementary File 1
