# Supplementary material for: Agelasine D Suppresses RANKL-Induced Osteoclastogenesis via Down-Regulation of c-Fos, NFATc1 and NF-κB
Source: Mar Drugs. 2014 Nov 24;12(11):5643–56. doi: 10.3390/md12115643 (PMC4245549; doi:10.3390/md12115643)
Supplement: Supplementary File 1 [file marinedrugs-12-05643-s001.pdf]

Supplementary Information

Figure S1. Purified TRAP-positive mononuclear osteoclast precursors were cultured with vehicle or AD (10 mM) in the presence of RANKL and M-CSF for 6 h and 12 h. Cells were stained with PE-conjugated anti-RANK or anti-c-Fms antibody at 4 ℃ for 10 min. After washing, the stained cells were analyzed by flow cytometry using a FACS Calibur with Cell Quest software.

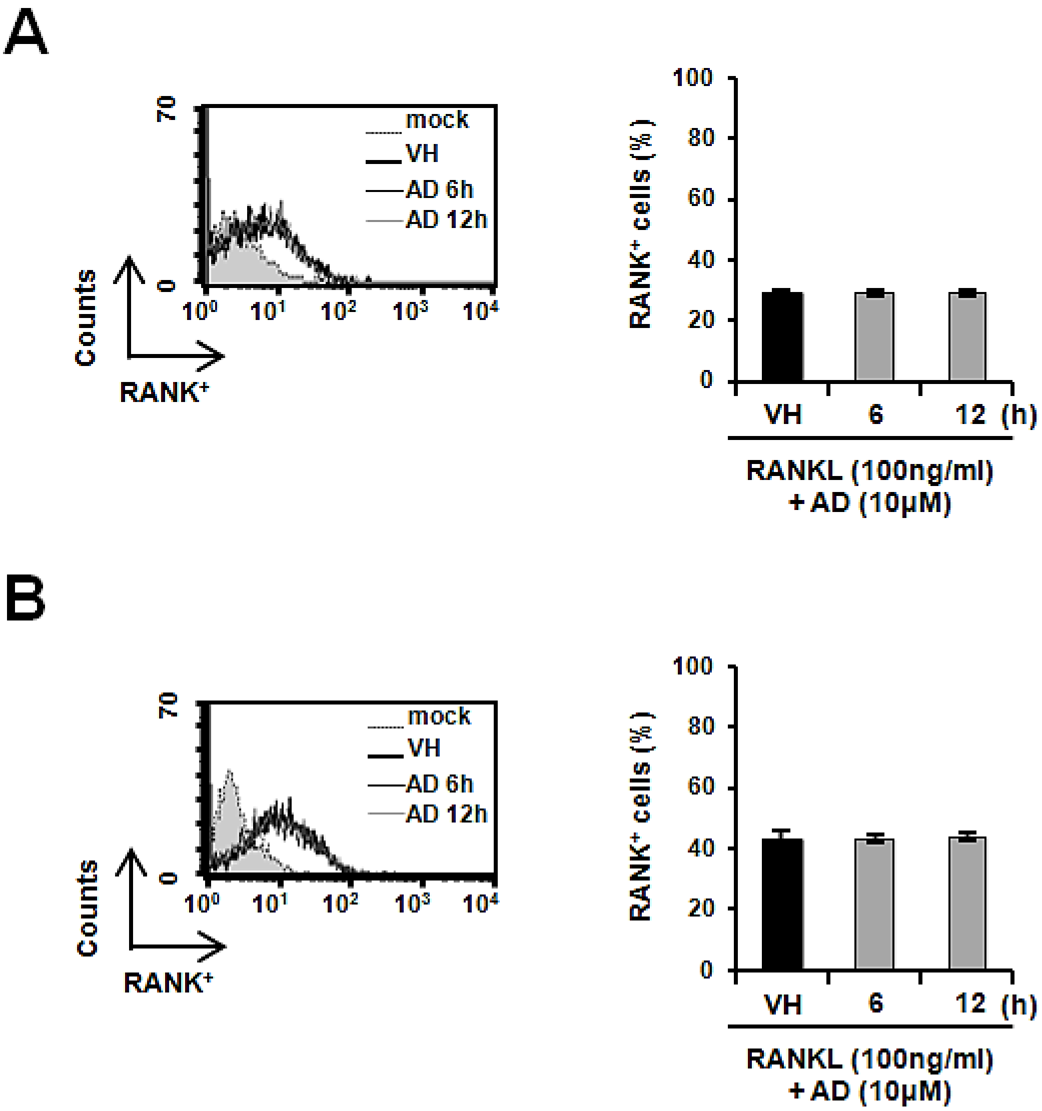

© 2014 by the authors; licensee MDPI, Basel, Switzerland. This article is an open access article distributed under the terms and conditions of the Creative Commons Attribution license (http://creativecommons.org/licenses/by/4.0/).
